# Efficacy and safety of ceftazidime/avibactam in patients with infections caused by β-lactamase-producing Gram-negative pathogens: a pooled analysis from the Phase 3 clinical trial programme

**DOI:** 10.1093/jac/dkad280

**Published:** 2023-09-13

**Authors:** Antoni Torres, Michele Wible, Margaret Tawadrous, Paurus Irani, Gregory G Stone, Alvaro Quintana, Dmitri Debabov, Margaret Burroughs, Patricia A Bradford, Marin Kollef

**Affiliations:** Servei de Pneumologia, Hospital Clinic, University of Barcelona, Villarroel 170, 08036, Barcelona, Spain; Pfizer, Collegeville, PA, USA; Hospital Business Unit, Pfizer, Groton, CT, USA; Hospital Business Unit, Pfizer, Tadworth, Surrey, UK; Hospital Business Unit, Pfizer, Groton, CT, USA; Hospital Business Unit, Pfizer, New York, NY, USA; Non-clinical Development Microbiology, AbbVie, Irvine, CA, USA; Global Pharmaceutical R&D, AbbVie, Madison, NJ, USA; Antimicrobial Development Specialists, LLC, Nyack, NY, USA; Division of Pulmonary & Critical Care Medicine, Institute of Clinical and Translational Sciences, Washington University School of Medicine, St Louis, MO, USA

## Abstract

**Objectives:**

This *post hoc* pooled analysis evaluated clinical and microbiological outcomes and safety in patients with infections caused by β-lactamase-producing Gram-negative pathogens across five Phase 3, randomized, controlled, multicentre trials of ceftazidime/avibactam in adults with complicated intra-abdominal infection (cIAI), complicated urinary tract infection (cUTI)/pyelonephritis and nosocomial pneumonia (NP), including ventilator-associated pneumonia (VAP).

**Methods:**

In each trial, RECLAIM/RECLAIM 3 (cIAI), REPRISE (cIAI/cUTI), RECAPTURE (cUTI) and REPROVE (NP, including VAP) patients were randomized 1:1 to IV ceftazidime/avibactam (plus metronidazole for patients with cIAI) or comparators (carbapenems in >97% patients) for 5–21 days. Clinical and microbiological responses at the test-of-cure visit were assessed for patients with ESBLs, and/or plasmidic and/or overexpression of chromosomal AmpC, and/or serine carbapenemases without MBLs identified in baseline Gram-negative isolates by phenotypic screening and molecular characterization in the pooled microbiological modified ITT (mMITT) population.

**Results:**

In total, 813 patients (ceftazidime/avibactam, *n *= 389; comparator, *n *= 424) had ≥1 β-lactamase-producing baseline pathogen identified, amongst whom 792 patients (ceftazidime/avibactam, *n* = 379; comparator, *n *= 413) had no MBLs. The most frequent β-lactamase-producing pathogens across treatment groups were *Escherichia coli* (*n *= 381), *Klebsiella pneumoniae* (*n *= 261) and *Pseudomonas aeruginosa* (*n *= 53). Clinical cure rates in the pooled non-MBL β-lactamase-producing mMITT population were 88.1% (334/379) for ceftazidime/avibactam and 88.1% (364/413) for comparators; favourable microbiological response rates were 76.5% (290/379) and 68.8% (284/413), respectively. The safety profile of ceftazidime/avibactam was consistent with previous observations.

**Conclusions:**

This analysis provides supportive evidence of the efficacy and safety of ceftazidime/avibactam in patients with infections caused by ESBLs, AmpC and serine carbapenemase-producing Gram-negative pathogens.

**Trial registration:**

NCT01499290; NCT01726023; NCT01644643; NCT01595438/NCT01599806; NCT01808092.

## Introduction

β-Lactamase enzymes are a major contributing factor to β-lactam resistance among Gram-negative bacteria. Isolates producing ESBLs, the most common β-lactamase enzymes, have increased in both healthcare and community settings over recent years; it is estimated that around 1.5 billion people are colonized with ESBL-producing Enterobacterales worldwide,^1^ with the most common ESBL producers being *Escherichia coli* and *Klebsiella* spp.^2,3^ ESBLs have been found to be present in 9.5% of *E. coli* and 16.3% of *Klebsiella pneumoniae* nosocomial isolates in Spain.^4^ Multiple β-lactam resistance mechanisms are also frequently identified in clinical isolates of *Pseudomonas aeruginosa*, of which many are considered to show ‘difficult-to-treat resistance’ (DTR) because of limited susceptibility to other antibiotics.^5^ Carbapenems are often used as first-line therapy for the treatment of infections caused by β-lactamase-producing Enterobacterales.^6–10^ However, with increased use of these agents, an increase in carbapenem-resistant Enterobacterales (CRE) has been observed over the past two decades.^11^

Ceftazidime/avibactam is a combination of the cephalosporin ceftazidime and the non-β-lactam β-lactamase inhibitor avibactam. The addition of avibactam to ceftazidime provides broad *in vitro* activity against many β-lactamases produced by Gram-negative organisms, including ESBLs, Ambler class A (e.g. *K. pneumoniae* carbapenemase), class C (AmpC) and some class D (e.g. OXA-48) serine carbapenemases, but not class B enzymes (MBLs).^12–14^ Ceftazidime/avibactam has demonstrated clinical and microbiological efficacy and safety in a range of serious infections, including those caused by MDR Gram-negative pathogens.^15–21^

Ceftazidime/avibactam is approved in Europe in adults and paediatric patients 3 months and older for the treatment of complicated intra-abdominal infection (cIAI) in combination with metronidazole, complicated urinary tract infection (cUTI)/pyelonephritis, hospital-acquired pneumonia [HAP; also referred to as nosocomial pneumonia (NP)] including ventilator-associated pneumonia (VAP), and for adult patients with bacteraemia associated with the above infections. It is also approved for infections caused by aerobic Gram-negative organisms in adults and paediatric patients with limited treatment options.^22^ In the USA, it is approved for cIAI, cUTI/pyelonephritis and hospital-acquired bacterial pneumonia/ventilator-associated bacterial pneumonia, in adults and children 3 months and older.^23^

An exploratory pooled analysis, described here, was performed to evaluate clinical and microbiological outcomes and safety in adults with infections caused by β-lactamase-producing (including ESBLs, AmpC and serine carbapenemases) Gram-negative pathogens across five ceftazidime/avibactam Phase III clinical trials.^15–19^

## Methods

### Ethics

This *post hoc* exploratory subset analysis was based on five Phase III ceftazidime/avibactam clinical trials for which primary efficacy and results have been previously reported.^15–19^ Ethics approval and informed patient consent were obtained for each trial, and all trials were conducted in accordance with the 1964 Declaration of Helsinki, and its later amendments.

### Patients and treatments

The five clinical trials enrolled patients with cUTI/pyelonephritis [RECAPTURE 1 and 2 (NCT01595438 and NCT01599806)],^19^ cIAI [RECLAIM 1 and 2 (NCT01499290) and RECLAIM 3 (NCT01726023)],^16,17^ cUTI or cIAI due to ceftazidime-resistant pathogens [REPRISE (NCT01644643)]^15^ or NP, including VAP [REPROVE (NCT01808092)].^18^ An overview of the five trials including patient populations, study designs and treatments is shown in Table S1 (available as Supplementary data at *JAC* Online).

Detailed methods, including patient inclusion/exclusion criteria, and overall efficacy and safety analyses have been reported previously.^15–19^ In brief, patients with cUTI, cIAI or NP, including VAP requiring IV therapy were randomized (1:1) to receive either ceftazidime/avibactam or comparator antibiotic (predominantly carbapenems) for 5–21 days. Ceftazidime/avibactam dosage was 2000/500 mg every 8 h (q8h) by 2 h IV infusions (adjusted for patients with renal impairment). A protocol amendment in the REPROVE (NP, including VAP) trial increased the ceftazidime/avibactam dose for patients with moderate-to-severe renal impairment at baseline (MSRIB; creatinine clearance 16–50 mL/min) by 50%. Patients with MSRIB receiving the original and increased dosage regimens were analysed separately in the primary analysis of REPROVE, and those receiving the original dosage regimen are excluded from the current pooled analysis. Metronidazole 500 mg (1 h IV infusion) q8h was given in conjunction with ceftazidime/avibactam for patients with cIAI. The comparator regimens were meropenem 1000 mg q8h (30 min IV infusion) in RECLAIM and REPROVE, doripenem 500 mg q8h (1 h IV infusion) in RECAPTURE 1 and 2, and best available therapy (BAT; consisting of a carbapenem-based regimen in ∼97% of patients) in REPRISE. Comparator doses were adjusted for renal function according to manufacturers’ recommendations.

### β-Lactamase profiling procedures

Baseline blood and/or infection site specimens for microbiological culture were obtained for all patients, including quantitative urine cultures for patients in RECAPTURE 1 and 2. For culture-positive specimens, species identification and susceptibility testing against study drugs was performed by the study sites’ local laboratories and confirmed by a central reference laboratory (Covance Central Laboratory Services, Indianapolis, IN, USA), according to CLSI and EUCAST criteria.^24,25^ Isolates sent to the central laboratory underwent a phenotypic MIC screen test according to CLSI criteria;^26^ those that were MIC screen positive (see Supplementary Methods) were candidates for molecular analyses, which included testing for ESBL and AmpC (plasmidic and overexpression of chromosomal AmpC) and/or carbapenemases. Molecular characterization was performed by JMI Laboratories (North Liberty, IA, USA). β-Lactamase genes were detected using a combination of Check-Points microarrays followed by PCR as described previously.^27–30^ Expression levels of AmpC were determined using a quantitative real-time PCR approach, with a threshold of up-regulation 5-fold above a reference value as described previously.^27^ If >1 isolate of the same species was obtained from the same patient that qualified for β-lactamase analysis, the molecular profiling data of all isolates were combined by including all β-lactamases identified and attributing them to a single isolate.

### Efficacy endpoints and assessments

In each trial, clinical response was assessed by investigators at the test-of-cure (TOC) visit [21–35 days (RECLAIM, RECAPTURE and REPROVE) or 7–10 days after last study treatment (REPRISE)]. Clinical cure was defined as complete resolution or significant improvement of signs and symptoms of the index infection with no further treatment required. Efficacy evaluations also included microbiological response at TOC. Favourable microbiological responses included eradication (absence of the causative pathogen from appropriately obtained specimens at the site of infection) and presumed eradication (repeat cultures were not performed, or were not clinically indicated in patients with clinical cure).

### Safety assessments

Safety assessments in each study included adverse event (AE) monitoring, vital signs measurements, physical examinations and clinical chemistry/laboratory tests up to the late follow-up visit (28–52 days post-randomization).

### Statistical methods

These exploratory subset analyses are based on the pooled population of patients with ≥1 β-lactamase-producing Gram-negative pathogen identified at baseline across the five ceftazidime/avibactam Phase III clinical trials. Such patients comprise a subset of the microbiological modified ITT (mMITT) populations in each trial, which included patients who met minimal disease inclusion criteria for the respective trials and had ≥1 Gram-negative pathogen identified at baseline.

There were no formal statistical hypotheses; all data were summarized descriptively. Baseline demographics, patient characteristics and clinical and microbiological response data were summarized for patients in the pooled mMITT population with ≥1 β-lactamase-producing Gram-negative pathogen identified by molecular characterization of MIC screen-positive baseline bacterial isolates as described above. Since neither ceftazidime/avibactam nor the comparator treatments are expected to be clinically effective against MBL-producing pathogens, patients with any MBL-producing pathogen(s) were excluded from the efficacy analyses. Clinical and microbiological responses at TOC were evaluated for ceftazidime/avibactam and comparators by indication and pooled across trials. Clinical cure and favourable microbiological response rates at TOC and associated 95% CIs calculated using the Jeffreys method^31,32^ are presented. Safety data were summarized for patients with ≥1 β-lactamase-producing Gram-negative pathogen in the safety population (randomized patients who received ≥1 dose of study drug).

## Results

### Patient population

**Figure 1.**
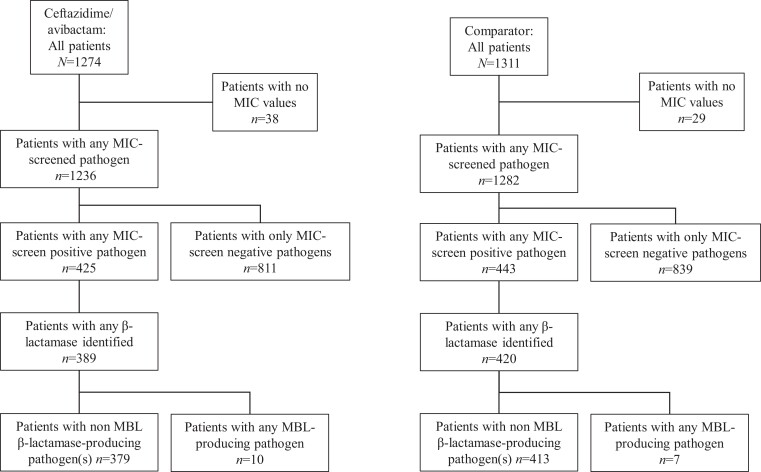
Overview of MIC screen test and molecular characterization outcomes to identify patients with β-lactamase-producing Gram-negative pathogens (pooled mMITT population). Patients could have ≥1 pathogen. Multiple isolates of the same species from the same patient are counted only once. Baseline isolates that met CLSI MIC criteria (see Supplementary Methods) were candidates for molecular analyses, which included testing for ESBL and AmpC and/or carbapenemases.

The pooled mMITT population included 2585 patients (ceftazidime/avibactam, *n *= 1274; comparator, *n *= 1311). An overview of MIC screen test and molecular characterization outcomes for baseline blood or infection site isolates from these patients is shown in Figure 1. Among those with a positive MIC screen test, 809 patients had ≥1 Gram-negative pathogen for which molecular analysis showed evidence of β-lactamase gene expression (or, in the case of *P. aeruginosa*, other β-lactam resistance mechanism): ceftazidime/avibactam [*n *= 389 (48.7%): cIAI, *n *= 104; cUTI, *n *= 231; NP/VAP, *n *= 54]; comparator [*n *= 420 (51.3%): cIAI, *n *= 130; cUTI, *n *= 232; NP/VAP, *n *= 58), of whom 792 patients (ceftazidime/avibactam, *n *= 379; comparator, *n *= 413) had no MBL-producing pathogen(s) identified; this subset of the pooled mMITT population was used in the current analyses of clinical and microbiological response.

### Patient characteristics and baseline pathogens

Baseline disease and demographic characteristics of patients with non-MBL β-lactamase-producing pathogens (Tables 1 and S2) were generally similar between the ceftazidime/avibactam and comparator arms, and were in line with the overall pooled ceftazidime/avibactam Phase II–III safety population.^20^ The most common primary diagnoses were cUTI without pyelonephritis, acute pyelonephritis and appendiceal perforation or peri-appendiceal abscess. Mean APACHE II scores were higher in patients with NP, including VAP, than in those with cIAI (Table S2); APACHE II score data were not collected for patients with cUTI/pyelonephritis.

Across the pooled treatment groups, >80% of patients had monomicrobial infections (Table 1). A summary of all Gram-negative pathogens isolated from patients with any β-lactamase-producing (non-MBL) pathogens is shown in Table S3, and by β-lactamase status in Table S4; the most frequently occurring pathogens were *E. coli*, isolated from 397 (50.1%) patients overall (381 β-lactamase-producing), *K. pneumoniae*, isolated from 278 (35.1%) patients (261 β-lactamase-producing), *P. aeruginosa*, isolated from 81 (10.2%) patients (53 β-lactamase-producing) and *Enterobacter cloacae*, isolated from 58 (7.3%) patients (54 β-lactamase-producing). The majority of β-lactamase-producing isolates expressed ≥2 enzymes, and ≥3 enzymes were identified across >75% of all β-lactamase-producing Enterobacterales isolates (Table S5). An overview of β-lactamase combinations is shown in Table 2. The most commonly identified combinations were ESBL + other class A + class D other than OXA-48, ESBL + other class A, and ESBL + class D other than OXA-48. Overall, 5 patients had class A carbapenemases and 11 had (class B) MBLs.

**Table 1.** Baseline demographic and disease characteristics in patients with β-lactamase-producing (non-MBL) Gram-negative pathogens identified at baseline (pooled mMITT population)

	Ceftazidime/avibactam (*N *= 379)	Comparator (*N *= 413)
Mean (SD) age, years	57.9 (17.6)	57.6 (17.9)
Age group, *n* (%), years		
≥18–45	96 (25.3)	103 (24.9)
46–64	123 (32.5)	139 (33.7)
65–74	90 (23.7)	92 (22.3)
≥75–≤90	70 (18.5)	79 (19.1)
Sex, *n* (%)		
Female	160 (42.2)	162 (39.2)
Male	219 (57.8)	251 (60.8)
Race, *n* (%)		
White	273 (72.0)	294 (71.2)
Black/African American	3 (0.8)	1 (0.2)
Asian	85 (22.4)	95 (23.0)
American Indian/Alaska Native	1 (0.3)	1 (0.2)
Other	17 (4.5)	22 (5.3)
Mean (SD) weight,^a^ kg	75.1 (18.9)	74.4 (17.8)
Mean (SD) BMI,^b^ kg/m^2^	26.6 (6.2)	26.4 (5.7)
Mean (SD) APACHE II score^c^	9.0 (5.6)	9.2 (6.2)
APACHE II score,^c^ *n* (%)		
<10	90 (23.7)	101 (24.5)
10–19	53 (14.0)	64 (15.5)
20–30	10 (2.6)	17 (4.1)
Missing/not done	226 (59.6)	231 (55.9)
CL_CR_ category, mL/min, *n* (%)		
≤30	7 (1.8)	8 (1.9)
31–50	44 (11.6)	42 (10.2)
51–80	135 (35.6)	133 (32.2)
≥81	192 (50.7)	229 (55.4)
Missing/not done	1 (0.3)	1 (0.2)
Primary diagnosis, *n* (%)		
Non-VAP	23 (6.1)	28 (6.8)
VAP	28 (7.4)	28 (6.8)
Acute pyelonephritis	100 (26.4)	114 (27.6)
cUTI without acute pyelonephritis	125 (33.0)	115 (27.8)
Traumatic perforation	3 (0.8)	1 (0.2)
Diverticular disease	5 (1.3)	7 (1.7)
Secondary peritonitis	13 (3.4)	10 (2.4)
Intra-abdominal abscess	12 (3.2)	15 (3.6)
Acute gastric and duodenal perforations	16 (4.2)	15 (3.6)
Cholecystitis	14 (3.7)	30 (7.3)
Appendiceal perforation or peri-appendiceal abscess	40 (10.6)	50 (12.1)
Infection type, *n* (%)		
Monomicrobial	307 (81.0)	339 (82.1)
Polymicrobial	72 (19.0)	74 (17.9)
2 pathogens	48 (12.7)	50 (12.1)
3 pathogens^d^	13 (3.4)	13 (3.1)
4 pathogens	6 (1.6)	10 (2.4)
≥ 5 pathogens	5 (1.3)	1 (0.2)

*N*, number of patients in the treatment arm; *n*, number of patients with specified characteristic.

^a^Weight ‘Not done/missing’ for one patient with NP, including VAP (ceftazidime/avibactam, *n *= 1).

^b^BMI ‘Not done/missing’ for three patients with cUTI (ceftazidime/avibactam, *n *= 2; comparator, *n *= 1) and one patient with NP, including VAP (ceftazidime/avibactam, *n *= 1).

^c^APACHE II score calculated programmatically using data obtained at the site and reported in the electronic case report form for patients with cIAI and NP, including VAP only. Data not collected for patients with cUTI. APACHE II score ‘Not done/missing’ for three patients with cIAI (ceftazidime/avibactam, *n *= 1; comparator, *n *= 2).

^d^A maximum of two uropathogens was allowed for study inclusion in the cUTI studies; however, one patient randomized to the ceftazidime/avibactam group in REPRISE presented with three pathogens at baseline (*Proteus mirabilis* in the urine culture and two anaerobes in the blood culture).

**Table 2.** β-Lactamase combinations in Gram-negative pathogens identified at baseline (pooled mMITT population)^a^

Category	β-Lactamase group combination^a^	Ceftazidime/avibactam, *n* (%) (*N *= 1274)	Comparator, *n* (%) (*N *= 1311)
Category I without Category II	Any	368 (28.9)	401 (30.6)
	ESBL + OXA-48 + other class A	0	1 (0.1)
	ESBL + OXA-48 + other class A + class D other than OXA-48	4 (0.3)	1 (0.1)
	ESBL + other class A	81 (6.4)	97 (7.4)
	ESBL + other class A + class D other than OXA-48	97 (7.6)	115 (8.8)
	ESBL + class A carbapenemase + other class A	1 (0.1)	2 (0.2)
	ESBL + class A carbapenemase + other class A + class D other than OXA-48	1 (0.1)	0
	ESBL + class C	2 (0.2)	5 (0.4)
	ESBL + class C + other class A	11 (0.9)	7 (0.5)
	ESBL + class C + other class A + class D other than OXA-48	11 (0.9)	16 (1.2)
	ESBL + class C + class D other than OXA-48	3 (0.2)	5 (0.4)
	ESBL + class D other than OXA-48	59 (4.6)	69 (5.3)
	OXA-48 + other class A	1 (0.1)	0
	Only ESBL	53 (4.2)	56 (4.3)
	Only class A carbapenemase	1 (0.1)	0
	Only class C	36 (2.8)	26 (2.0)
	Class A carbapenemase + other class A	2 (0.2)	4 (0.3)
	Class C + other class A	13 (1.0)	14 (1.1)
	Class C + other class A + class D other than OXA-48	3 (0.2)	4 (0.3)
	Class C + class D other than OXA-48	6 (0.5)	3 (0.2)
Any Category II	Any	10 (0.8)	7 (0.5)
	ESBL + class B + other class A	1 (0.1)	4 (0.3)
	ESBL + class B + other class A + class D other than OXA-48	1 (0.1)	0
	ESBL + class C + class B + other class A	1 (0.1)	1 (0.1)
	Only class B	5 (0.4)	0
	Class B + other class A	1 (0.1)	2 (0.2)
	Class B + class D other than OXA-48	3 (0.2)	0
Only Category III	Any	11 (0.9)	19 (1.4)
	Only other class A	8 (0.6)	12 (0.9)
	Only class D other than OXA-48	3 (0.2)	5 (0.4)
	Other class A + class D other than OXA-48	0	2 (0.2)

*N*, number of patients in the treatment arm with a pathogen of the specified type; *n*, number of patients with pathogen classified in the β-lactamase category.

^a^Category I β-lactamase group combinations include pathogens with genes encoding β-lactamases expected to be inhibited by the combination of ceftazidime ± avibactam (OXA-48, which confers resistance to carbapenems but not ceftazidime is included, since OXA-48 producers frequently produce ESBLs that additionally make them resistant to ceftazidime). Category II combinations include pathogens with genes encoding any β-lactamases not expected to be inhibited by ceftazidime/avibactam or comparator treatments (i.e. class B carbapenemases). Category III combinations include pathogens with genes encoding β-lactamases inhibited by avibactam for which avibactam is not expected to be of additional clinical benefit because they do not hydrolyse ceftazidime or carbapenems. Patients could have ≥1 pathogen and could have pathogens in ≥1 β-lactamase group combination.

The specific β-lactamase enzymes and resistance mechanisms identified are shown in Table S6; across all β-lactamase-producing isolates the most frequently identified enzymes were CTX-M-like (624 isolates), CTX-M-15-like (470 isolates) and OXA-like (387 isolates). AmpC overexpression was identified in 76 isolates of *E. cloaca*e (37), *P. aeruginosa* (34) and *Citrobacter freundii* complex (5).

In patients with β-lactamase-producing (non-MBL) pathogens from any source, 38 had positive blood cultures at baseline (bacteraemia; 14 and 24 in the ceftazidime/avibactam and comparator groups, respectively), of whom 26 patients (9 and 17, respectively) had β-lactamase-producing strains (*K. pneumoniae*, *E. coli*, *E. cloacae* and *Providencia rettgeri*) isolated from blood cultures.

Across all β-lactamase-producing (non-MBL) baseline pathogens, 99.9% of Enterobacterales and 76.5% of *P. aeruginosa* isolates were susceptible to ceftazidime/avibactam (MIC ≤8 mg/L), based on CLSI and EUCAST breakpoints^24,25^ and ≥90% of Enterobacterales isolates were inhibited at ≤1 mg/L (Table S7). β-Lactamase-producing (non-MBL) isolates were generally susceptible to the relevant comparators including BAT across the respective trials.

### Efficacy evaluation

Clinical cure rates at TOC for patients with β-lactamase-producing (non-MBL) baseline pathogens are summarized in Table 3. Clinical cure rates at TOC for all indications combined were 88.1% (334/379; 95% CI 84.6–91.9) for ceftazidime/avibactam and 88.1% (364/413; 95% CI 84.8–91.0) for comparators. Respective clinical cure rates at TOC were highest for patients with cUTI (91.1% and 92.1%), followed by those with cIAI (85.4% and 85.9%), and lowest for patients with NP, including VAP (80.4% and 76.8%).

**Table 3.** Clinical and microbiological responses at TOC in patients with β-lactamase-producing (non-MBL) Gram-negative pathogens identified at baseline (mMITT population)

	cIAI		cUTI/pyelonephritis		NP, including VAP		All indications combined	
Patients, *n* (%)	Ceftazidime/avibactam + metronidazole (*n* = 103)	Comparator (*n* = 128)	Ceftazidime/avibactam (*n* = 225)	Comparator (*n* = 229)	Ceftazidime/avibactam (*n* = 51)	Meropenem (*n* = 56)	Ceftazidime/avibactam (*n* = 379)	Comparator (*n* = 413)
Clinical response								
* *Clinical cure	88 (85.4)	110 (85.9)	205 (91.1)	211 (92.1)	41 (80.4)	43 (76.8)	334 (88.1)	364 (88.1)
* *95% CI^a^	77.7–91.2	79.1–91.1	86.9–94.3	88.1–95.1	68.0–89.4	64.6–86.3	84.6–91.1	84.8–91.0
* *Clinical failure	7 (6.8)	3 (2.3)	4 (1.8)	9 (3.9)	8 (15.7)	10 (17.9)	19 (5.0)	22 (5.3)
* *Indeterminate	8 (7.8)	15 (11.7)	16 (7.1)	9 (3.9)	2 (3.9)	3 (5.4)	26 (6.9)	27 (6.5)
Microbiological response								
* *Favourable	88 (85.4)	110 (85.9)	172 (76.4)	143 (62.4)	30 (58.8)	31 (55.4)	290 (76.5)	284 (68.8)
* *95% CI^a^	77.7–91.2	79.1–91.1	70.6–81.6	56.0–68.5	45.1–71.5	42.3–67.8	72.1–80.6	64.–73.1
* *Unfavourable	7 (6.8)	3 (2.3)	36 (16.0)	74 (32.3)	19 (37.3)	22 (39.3)	62 (16.4)	99 (24.0)
* *Indeterminate	8 (7.8)	15 (11.7)	17 (7.6)	12 (5.2)	2 (3.9)	3 (5.4)	27 (7.1)	30 (7.3)

^a^Calculated using the Jeffreys method.^31,32^

Clinical cure rates by baseline pathogen for patients in the β-lactamase-producing (non-MBL) population (Table 4 and Table S8) were generally comparable between treatment groups and similar to the overall results for the population. Clinical cure rates in subgroups of potential clinical interest (Figure S1) were generally similar between ceftazidime/avibactam and comparators, with overlapping 95% CIs within each subgroup.

**Table 4.** Clinical cure rates at TOC by baseline pathogen in patients with β-lactamase-producing (non-MBL) Gram-negative pathogens identified at baseline (mMITT population)^a^

	cIAI		cUTI/pyelonephritis		NP, including VAP		All indications combined	
Patients, *n*/*N* (%)	Ceftazidime/avibactam + metronidazole (*n *= 103)	Comparator (*n *= 128)	Ceftazidime/avibactam (*n *= 225)	Comparator (*n *= 229)	Ceftazidime/avibactam (*n *= 51)	Meropenem (*n *= 56)	Ceftazidime/avibactam (*n *= 379)	Comparator (*n *= 413)
Enterobacterales (all)	86/101 (85.1)	109/127 (85.8)	191/208 (91.1)	211/229 (92.1)	38/47 (80.9)	43/56 (76.8)	315/356 (88.5)	347/395 (87.8)
* C. freundii* complex	0/1 (0.0)	2/2 (100)	6/6 (100)	2/2 (100)	0/0	0/0	6/7 (85.7)	6/7 (85.7)
* Enterobacter aerogenes*	0/0	1/1 (100)	1/1 (100)	0/0	3/4 (75.0)	2/2 (100)	4/5 (80.0)	3/3 (100)
* E. cloacae*	6/7 (85.7)	6/8 (75.0)	10/13 (76.9)	11/13 (84.6)	10/10 (100)	3/7 (42.9)	26/30 (86.7)	20/28 (71.7)
* E. coli*	62/75 (82.7)	83/96 (86.5)	97/106 (91.5)	94/105 (89.5)	4/6 (66.7)	6/9 (66.7)	163/187 (87.2)	183/210 (87.1)
* K. pneumoniae*	23/27 (85.2)	16/21 (76.2)	71/74 (95.9)	89/94 (94.7)	24/30 (80.0)	25/32 (78.1)	118/131 (90.1)	130/147 (88.4)
* P. mirabilis*	3/3 (100)	3/3 (100)	7/9 (77.8)	6/6 (100)	6/6 (100)	4/5 (80.0)	16/18 (88.9)	13/14 (92.9)
* Serratia marcescens*	2/2 (100)	1/1 (100)	0/0	3/3 (100)	2/3 (66.7)	4/5 (80.0)	4/5 (80.0)	8/9 (88.9)
Other Gram-negative pathogens (all)	7/11 (63.6)	12/12 (100)	15/18 (83.3)	10/10 (100)	12/15 (80.0)	15/21 (76.8)	34/44 (77.3)	37/43 (86.0)
* P. aeruginosa*	3/7 (42.9)	12/12 (100)	15/18 (83.3)	10/10 (100)	12/15 (80.0)	14/19 (73.7)	30/40 (75.0)	36/41 (87.8)

Patients could have ≥1 pathogen. Multiple isolates of the same species from the same patient are counted only once.

^a^For pathogens with *n *≥ 5 cases in either treatment group (all indications combined).

Favourable microbiological responses at TOC for patients with β-lactamase-producing (non-MBL) baseline pathogens across all indications combined were achieved in 76.5% (290/379) patients in the ceftazidime/avibactam group and 68.8% (284/413) in the comparator group. Favourable microbiological response rates were highest in patients with cIAI and lowest in those with NP, including VAP. Favourable microbiological response rates at TOC by baseline pathogen are summarized in Table S9.

### Safety evaluation

The frequencies and patterns of AEs, including serious AEs (SAEs), severe AEs and AEs leading to discontinuation of study drug in patients with β-lactamase-producing Gram-negative pathogens (pooled safety population) were similar between treatment groups overall and within each indication (Table 5). In both treatment groups, the most common AEs were diarrhoea, nausea and headache. SAEs were reported for 21 (5.4%) and 24 (5.7%) patients in the ceftazidime/avibactam and comparator groups, respectively (Table 5). SAEs occurring in >1 patient in either treatment group comprised pneumonia (ceftazidime/avibactam, *n* = 1; comparator, *n* = 3), atrial fibrillation (comparator, *n* = 3), cardiac failure (ceftazidime/avibactam, *n* = 2), respiratory failure (comparator, *n* = 2), and acute kidney injury (comparator, *n* = 2). Overall, AEs with an outcome of death occurred in 6 (1.5%) patients in the ceftazidime/avibactam group, and 5 (1.3%) patients in the comparator group.

**Table 5.** Overview of AEs in patients with β-lactamase-producing Gram-negative pathogens identified at baseline (safety population)

	cIAI		cUTI/pyelonephritis		NP, including VAP		All indications combined	
Patients, *n* (%)	Ceftazidime/avibactam + metronidazole (*n *= 104)	Comparator (*n *= 134)	Ceftazidime/avibactam (*n *= 231)	Comparator (*n *= 232)	Ceftazidime/avibactam (*n *= 54)	Meropenem (*n *= 58)	Ceftazidime/avibactam (*n *= 389)	Comparator (*n *= 424)
Any AE	45 (43.3)	55 (41.0)	78 (33.8)	77 (33.2)	38 (70.4)	39 (67.2)	161 (41.4)	171 (40.3)
Any AE with outcome of death	2 (1.9)	1 (0.7)	3 (1.3)	2 (0.9)	1 (1.9)	2 (3.4)	6 (1.5)	5 (1.2)
Any SAE	7 (6.7)	12 (9.0)	9 (3.9)	6 (2.6)	5 (9.3)	6 (10.3)	21 (5.4)	24 (5.7)
AE leading to discontinuation of study drug	3 (2.9)	1 (0.7)	3 (1.3)	2 (0.9)	1 (1.9)	1 (1.7)	7 (1.8)	4 (0.9)
Any AE of severe intensity	6 (5.8)	11 (8.2)	4 (1.7)	7 (3.0)	4 (7.4)	7 (12.1)	14 (3.6)	25 (5.9)
AEs occurring in ≥2% patients in either treatment group (all indications) by MedDRA preferred term								
Diarrhoea	5 (4.8)	8 (6.0)	6 (2.6)	10 (4.3)	10 (18.5)	13 (22.4)	21 (5.4)	31 (7.3)
Nausea	14 (13.5)	8 (6.0)	3 (1.3)	12 (5.2)	4 (7.4)	0 (0.0)	21 (5.4)	20 (4.7)
Headache	5 (4.8)	4 (3.0)	9 (3.9)	13 (5.6)	0 (0.0)	0 (0.0)	14 (3.6)	17 (4.0)
Vomiting	4 (3.8)	5 (3.7)	2 (0.9)	1 (0.4)	5 (9.3)	7 (12.1)	11 (2.8)	13 (3.1)
Pyrexia	6 (5.8)	7 (5.2)	3 (1.3)	3 (1.3)	2 (3.7)	3 (5.2)	11 (2.8)	13 (3.1)
Dyspepsia	0 (0.0)	4 (3.0)	1 (0.4)	6 (2.6)	0 (0.0)	0 (0.0)	1 (0.3)	10 (2.4)
Anaemia	2 (1.9)	1 (0.7)	1 (0.4)	2 (0.9)	6 (11.1)	4 (6.9)	9 (2.3)	7 (1.7)
Pneumonia	1 (1.0)	4 (3.0)	0 (0.0)	1 (0.4)	1 (1.9)	4 (6.9)	2 (0.5)	9 (2.1)
Hypokalaemia	2 (1.9)	0 (0.0)	1 (0.4)	3 (1.3)	5 (9.3)	5 (8.6)	8 (2.1)	8 (1.9)

MedDRA, Medical Dictionary for Regulatory Activities.

## Discussion

β-Lactamase-producing organisms are pervasive worldwide,^1,33^ and ESBLs represent the most common cause of resistance to third-generation cephalosporins.^3^ Currently, the largest group of ESBLs is CTX-Ms,^34^ which have predominated in recent years over TEM and SHV variants, largely as a result of clonal expansion.^35^ In line with these observations, approximately 30% of patients in the pooled Phase III mMITT population had ≥1 β-lactamase-producing Gram-negative pathogen identified following a positive MIC screen test, and the most frequently identified enzymes in the current analysis were CTX-M-like and CTX-M-15-like; most β-lactamase-producing isolates included in the analysis harboured multiple (i.e. ≥2) enzymes.

The findings of this pooled exploratory subset analysis provide supportive evidence of the effectiveness and safety of ceftazidime/avibactam in patients with cIAI, cUTI/pyelonephritis or NP, including VAP, caused by ESBL-, AmpC- and serine carbapenemase-producing Gram-negative pathogens across five Phase III multinational prospective studies. No new safety issues were identified for ceftazidime/avibactam. The most common AEs across both treatment groups were diarrhoea, nausea and headache, which are known adverse drug reactions for ceftazidime/avibactam.^20,22,23^ The safety profile of ceftazidime/avibactam was in line with the overall safety profile for ceftazidime/avibactam in adults, with similar rates of AEs (41.4% in the current analysis, and 49.2% in the overall Phase II–III safety population), SAEs (5.4% and 8.7%, respectively) and AEs with an outcome of death (1.5% and 2.0%, respectively).^20^ The ceftazidime/avibactam safety profile in this analysis was also consistent with the established safety profile of ceftazidime monotherapy.

Ceftazidime/avibactam demonstrated non-inferiority versus carbapenem comparators in each of the four individual double-blind Phase III trials, and clinical and microbiological efficacy similar to BAT (of which 97% were carbapenems) in the open-label REPRISE trial.^15–19^ In the current analysis, which pooled data for patients with β-lactamase-producing Gram-negative pathogens across these five trials, clinical cure and favourable microbiological response rates at TOC were generally high, and similar for ceftazidime/avibactam and comparators within each indication and across all indications combined. Across indications, 88.1% of patients in both treatment groups achieved clinical cure at TOC; within indications, favourable clinical and microbiological response rates at TOC were generally consistent with those in the individual trials.^15–19^ Of note, patients with cIAI and moderate or severe renal impairment in RECLAIM 1 and 2 received a lower modified dose of ceftazidime/avibactam than is currently approved. This underdosing was associated with reduced efficacy within the renal impairment subgroup^16^ and is also reflected within the current analysis. Favourable per-pathogen microbiological response rates were generally higher for patients with infections involving Enterobacterales, and were more variable for those involving *P. aeruginosa*, for both ceftazidime/avibactam and comparator; this is consistent with the *in vitro* susceptibility profiles of the study isolates and the overall results within each trial, as well as other analyses.^21,36–38^ For example, Isler *et al.*^39^ conducted a meta-analysis of ceftazidime/avibactam for the treatment of infections caused by ESBL/AmpC-producing Enterobacterales across the Phase II and III ceftazidime/avibactam trials (in contrast, we used Phase III trial data only, and included all Gram-negative pathogens). Consistent with the present analysis, Isler *et al.*^39^ reported clinical cure rates at TOC of 91% for ceftazidime/avibactam and 89% for the comparator group in patients with ESBL/AmpC-producing Enterobacterales.

Early initiation of appropriate antimicrobial therapy for infections caused by ESBL-producing pathogens has been shown to reduce morbidity and mortality.^11,40,41^ A number of agents that show activity against ESBL-producing pathogens have recently become available for treatment of serious infections and/or those with limited treatment options. In clinical trials, the siderophore cephalosporin cefiderocol was non-inferior to carbapenems for the treatment of cUTI and NP, including cases caused by ESBL-producing pathogens.^42,43^ However, increased mortality was reported in patients treated with cefiderocol (compared with those treated with BAT), predominantly in patients with *Acinetobacter* spp. NP or bloodstream infections.^44^ The carbapenem/β-lactamase inhibitor combinations meropenem/vaborbactam and imipenem/cilastatin/relebactam have both demonstrated good *in vitro* activity against ESBL-producing pathogens;^45–47^ however, there are limited clinical data available from patients documented as having infections caused by ESBL producers. Finally, studies have suggested that the cephalosporin/β-lactamase inhibitor combination ceftolozane/tazobactam may be a valid treatment option in patients with serious infections caused by ESBL-producing Enterobacterales.^48–50^ Definitive therapy for ESBL infections typically involves carbapenems,^8,51,52^ and the use of a carbapenem-sparing regimen of piperacillin/tazobactam in the MERINO trial was shown to be less effective than meropenem at preventing 30 day mortality in patients with ceftriaxone-non-susceptible *E. coli* or *K. pneumoniae* bacteraemia.^53^ Nonetheless, carbapenem-sparing strategies remain of interest and are currently being explored in other studies, including the MERINO 3 trial, evaluating the effect of ceftolozane/tazobactam versus meropenem on 30 day mortality in a similar patient population.^54^ In addition to consideration of initial empirical use of carbapenem-sparing antimicrobial therapies in patients infected with ESBL producers, with potential for switch to appropriate targeted therapy once pathogens are identified, there may be a role for tailoring empirical use of cephalosporin/β-lactamase inhibitor to specific clinical situations, e.g. in patients suspected to be infected not only by ESBL, Amp C and/or serine carbapenemase producers, but also those with suspected carbapenem-resistant *Pseudomonas* spp., as well as in patients with carbapenem intolerance.^39^

Multinational surveillance studies have consistently reported that ceftazidime/avibactam demonstrates potent *in vitro* activity comparable to carbapenems against ESBL- and AmpC-producing Enterobacterales isolates across multiple geographical regions.^55–57^ A recent WGS analysis of isolates collected from patients with a range of infection types showed ceftazidime/avibactam to be active against 100% of *E. coli, K. pneumoniae* and *E. cloacae* isolates carrying common ESBL-encoding genes, including *bla*_CTX-M_ or *bla*_SHV_.^58^ Ceftazidime/avibactam has also been reported to be the most effective carbapenem alternative against ESBL-producing *E. coli* and *K. pneumoniae* isolated from patients with UTI, including those with bacteraemic infections.^59^

Limitations of this exploratory *post hoc* descriptive analysis include the small numbers of patients in some subgroups, with fewer patients with confirmed β-lactamase-producing pathogens in the more severely ill NP (including VAP) subgroup, and more patients in the cUTI/acute pyelonephritis subgroup. Moreover, among patients with cIAI, mean APACHE II scores were <10, and among patients with NP/VAP, mean APACHE II scores were <20, higher scores indicating more severe clinical status (APACHE II scores were not assessed in patients with cUTI). Caution is therefore required in the generalization of these results to the most severely ill patients. As with all such exploratory subgroup analyses, the results cannot be considered as robust as those from prespecified analyses with appropriate stratification for baseline factors, and as such should be interpreted with caution. The lack of baseline stratification for β-lactamase status and exclusion of patients with septic shock, hepatic failure, renal failure or immunosuppression, and lack of assessments at an early timepoint (e.g. 5 days), are additional limitations arising from the design of the Phase III studies. Moreover, the design of the clinical trials, in which ceftazidime/avibactam and comparators were administered empirically for a defined duration, was necessary and appropriate to provide evidence of clinical effectiveness and safety; however, it does not reflect real-world practice, in which empirical therapy is often de-escalated following pathogen identification and susceptibility results. Nevertheless, the results provide supporting evidence of the efficacy and safety of ceftazidime/avibactam in patients with cIAI, cUTI/pyelonephritis or NP, including VAP with involvement of β-lactamase-producing Gram-negative pathogens, and ceftazidime/avibactam is recognized as a preferred treatment option for many infections caused by CRE and DTR *P. aeruginosa*.^5^ Real-world studies and ongoing clinical experience will provide additional insights regarding the utility of ceftazidime/avibactam in this setting.

## Supplementary Material

dkad280_Supplementary_Data

## Transparency declarations

A.T. and M.K. received institutional research funding from AstraZeneca for conducting clinical studies included in this analysis. AstraZeneca’s rights to ceftazidime/avibactam were acquired by Pfizer in December 2016. Ceftazidime/avibactam is being co-developed by Pfizer and AbbVie (following its acquisition of Allergan). M.W., M.T., G.G.S, P.I. and A.Q. are employees of Pfizer and hold Pfizer shares/and or stock options. D.D. and M.B. are employees of AbbVie (following its acquisition of Allergan). P.A.B. is a former employee of AstraZeneca. M.K. is supported by the Barnes-Jewish Hospital Foundation.

## Data sharing

Upon request, and subject to certain criteria, conditions and exceptions, (see https://www.pfizer.com/science/clinical-trials/trial-data-and-results for more information), Pfizer will provide access to individual de-identified participant data from Pfizer-sponsored global interventional clinical studies conducted for medicines, vaccines and medical devices (i) for indications that have been approved in the USA and/or EU; or (ii) in programmes that have been terminated (i.e. development for all indications has been discontinued). Pfizer will also consider requests for the protocol, data dictionary and statistical analysis plan. Data may be requested from Pfizer trials 24 months after study completion. The de-identified participant data will be made available to researchers whose proposals meet the research criteria and other conditions, and for which an exception does not apply, via a secure portal. To gain access, data requestors must enter into a data access agreement with Pfizer.

## Supplementary data

Figure S1 and Tables S1 to S9 are available as Supplementary data at *JAC* Online.
