# Supplementary material for: Efficacy and safety of ceftazidime/avibactam in patients with infections caused by β-lactamase-producing Gram-negative pathogens: a pooled analysis from the Phase 3 clinical trial programme
Source: J Antimicrob Chemother. 2023 Sep 13;78(11):2672–82. doi: 10.1093/jac/dkad280 (PMC11157139; doi:10.1093/jac/dkad280)
Supplement: dkad280_Supplementary_Data [file dkad280_supplementary_data.pdf]

Supplementary Data

Efficacy and safety of ceftazidime/avibactam in patients with infections caused by β-lactamase-producing Gram-negative pathogens: a pooled analysis from the phase 3 clinical trial programme

Antoni Torres[1]*, Michele Wible[2], Margaret Tawadrous[3], Paurus Irani[4], Gregory G. Stone[3], Alvaro Quintana[5], Dmitri Debabov[6], Margaret Burroughs[7], Patricia A. Bradford[8], and Marin Kollef[9]

[1]Servei de Pneumologia, Hospital Clinic, University of Barcelona, Barcelona, Spain; [2]Pfizer, Collegeville, PA, USA; [3]Pfizer, Groton, CT, USA; [4]Pfizer, Tadworth, Surrey, UK; [5]Pfizer, New York, NY, USA; [6]AbbVie, Irvine, CA, USA; [7]AbbVie, Madison, NJ, USA; [8]Antimicrobial Development Specialists, LLC. Nyack, NY, USA; [9]Washington University School of Medicine, St Louis, MO, USA

Supplementary Methods

Selection of isolates for molecular analysis

Baseline isolates that met the following Clinical and Laboratory Standards Institute (CLSI) MIC criteria[1] were candidates for molecular analyses, which included testing for ESBL and AmpC and/or carbapenemases.

ESBL and AmpC testing

- *E. coli*, *Klebsiella pneumoniae*, and *K. oxytoca* with cefpodoxime MICs of ≥8 mg/L or ceftazidime, aztreonam, cefotaxime, or ceftriaxone MICs of ≥2 mg/L.
- *P. mirabilis* with cefpodoxime, ceftazidime, or cefotaxime MICs of ≥2 mg/L.
- *Serratia* spp., *Providencia* spp., *Citrobacter* spp., and *Enterobacter* spp. with cefepime, ceftazidime, or ceftriaxone MICs of ≥2 mg/L.
- Non-fermentative Gram-negative strains with ceftazidime MICs of ≥16 mg/L.

Carbapenemase testing

- *Enterobacterales* with meropenem MICs of ≥2 mg/L.
- Non-fermentative Gram-negative strains with meropenem MICs of ≥8 mg/L.
- The selected *Enterobacterales* strains also had transcription levels of bla_{AmpC} determined.

AmpC expression

- *E. coli* and *Klebsiella* spp. strains had the expression levels of bla_{AmpC} determined only when other ESBL screening results were negative Additionally, the transcription levels of the chromosomally located bla_{AmpC} among *Serratia* spp.,

Providencia spp., *Citrobacter* spp., and *Enterobacter* spp. were determined.

- The transcription levels of bla_{AmpC} were considered significant (upregulated) when the relative quantification was >5-fold the reference strain.
- Non-fermentative Gram-negative organisms with ceftazidime MIC values of ≥16 mg/L had transcription levels of chromosomally encoded bla_{AmpC} determined.

Supplementary Results

Table S1. Overview of ceftazidime/avibactam phase 3 clinical trials included in the pooled analysis

Trial; clinicaltrials.gov registration number	Patient population	Study design and duration of treatment	Ceftazidime/avibactam dosage regimen	Comparator dosage regimen	Number of patients			
					Safety population[a]		mMITT population[b]	
					Ceftazidime-avibactam	Comparator	Ceftazidime-avibactam	Comparator
RECLAIM 1/2; NCT01499290[2]	Age 18–90[b] years with cIAI requiring surgical intervention or percutaneous drainage within 24 h	Prospective, randomized, double-blind<br><br>5–14 days	2000-500 mg 2-h IV infusion q8h + metronidazole 500 mg 1-h IV infusion q8h[d]	Meropenem 1000 mg 30-min IV infusion q8h[d]	529	529	413	410
RECLAIM 3; NCT01726023[3]	Age 18–90 years with cIAI requiring surgical intervention	Prospective, randomized, double-blind<br><br>5–14 days	2000-500 mg 2-h IV infusion q8h + metronidazole 500 mg 1-h IV infusion q8h[d]	Meropenem 1000 mg, 30-min IV infusion q8h[d]	215	217	143	152
REPRISE; NCT01644643[4]	Age 18–90 years with cIAI or cUTI/ pyelonephritis caused by ceftazidime-resistant Gram-negative pathogens	Prospective, randomized, open-label<br><br>5–21 days	2000-500 mg 2-h IV infusion q8h + for patients with cIAI 500 mg metronidazole 60-min IV infusion q8h[d]	BAT: determined before randomization based on the investigator's standard of care and the local label[e] recommendation	12 (cIAI)<br>152 (cUTI)	15 (cIAI)<br>153 (cUTI)	10 (cIAI)<br>144 (cUTI)	11 (cIAI)<br>137 (cUTI)
RECAPTURE 1/2; NCT01595438; NCT01599806[5]	Age 18–90 years with cUTI/ pyelonephritis	Prospective, randomized, double-blind	2000-500 mg 2-h IV infusion q8h	Doripenem 500 mg, 60-min IV infusion q8h	511	509	393	417

		5–14 days (with optional switch to open-label oral therapy after ≥5 days of IV therapy)						
REPROVE; NCT01808092[6]	Age 18–90 years with NP, including VAP[c]	Prospective, randomized, double-blind<br><br>7–14 days	2000-500 mg 2-h IV infusion q8h	Meropenem 1000 mg, 30-min IV infusion q8h	405	403	171	184

[a]Randomized and received at least one dose of study treatment.

[b]18–65 years in India.

[c]18–65 years in India.

[d]Doses of ceftazidime/avibactam and meropenem were reduced in patients with moderate renal impairment (estimated creatinine clearance 31–≤50 mL/min) to 1000-250 mg q12h and 1000 mg q12h, respectively. No dose adjustments were required for metronidazole.

[e]Preferred BAT options, determined before randomization, for cIAI and cUTI were 5–21 days' treatment with meropenem, imipenem, doripenem, colistin, and (for cIAI only) tigecycline, administered intravenously; 97% of patients in BAT group received a carbapenem, the majority as monotherapy

[f]For patients treated with BAT, dose adjustments for patients with renal impairment were to be followed as per the locally accepted standard of care and local label.

Doses of ceftazidime/avibactam and meropenem in REPROVE were adjusted in patients (MSRIB; estimated creatinine clearance 16–≤50 mL/min). A protocol amendment in Year 3 (of 4) of the study increased the ceftazidime/avibactam dose for patients with MSRIB by 50%. Patients with MSRIB receiving the original dose regimens were analysed separately in the main study, and are excluded from these analyses.

BAT, best available therapy; cIAI, complicated intra-abdominal infection; cUTI, complicated urinary tract infection; IV, intravenous; mMITT, microbiological modified intent-to-treat; MSRIB, moderate-to-severe renal impairment at baseline; NP, nosocomial pneumonia; q8h, every 8 h; q12h, every 12 h; VAP, ventilator-associated pneumonia.

Table S2. Baseline demographic and disease characteristics according to indication in patients with β-lactamase-producing (non MBL) Gram-negative pathogens identified at baseline (mMITT population)

	cIAI		cUTI/pyelonephritis		NP, including VAP	
	Ceftazidime/avibactam + metronidazole (*n*=103)	Comparator (*n*=128)	Ceftazidime/avibactam (*n*=225)	Comparator (*n*=229)	Ceftazidime/avibactam (*n*=51)	Meropenem (*n*=56)
Mean (SD) age, years	47.9 (17.0)	48.0 (17.8)	61.0 (16.4)	61.5 (14.8)	64.3 (17.7)	63.6 (20.9)
Age group (years), *n* (%)						
≥18–45	42 (40.8)	56 (43.8)	47 (20.9)	34 (14.8)	7 (13.7)	13 (23.2)
46–64	43 (41.7)	45 (35.2)	64 (28.4)	85 (37.1)	16 (31.4)	9 (16.1)
65–74	13 (12.6)	16 (12.5)	64 (28.4)	68 (29.7)	13 (25.5)	8 (14.3)
≥75–≤90	5 (4.9)	11 (8.6)	50 (22.2)	42 (18.3)	15 (29.4)	26 (46.4)
Sex, *n* (%)						
Female	42 (40.8)	47 (36.7)	104 (46.2)	100 (43.7)	14 (27.5)	15 (26.8)
Male	61 (59.2)	81 (63.3)	121 (53.8)	129 (56.3)	37 (72.5)	41 (73.2)
Race, *n* (%)						
White	40 (38.8)	65 (50.8)	202 (89.8)	203 (88.6)	31 (60.8)	26 (46.4)
Black/African American	1 (1.0)	0 (0.0)	2 (0.9)	1 (0.4)	0 (0.0)	0 (0.0)
Asian	60 (58.3)	63 (49.2)	5 (2.2)	5 (2.2)	20 (39.2)	27 (48.2)
American Indian/Alaska Native	1 (1.0)	0 (0.0)	0 (0.0)	1 (0.4)	0 (0.0)	0 (0.0)
Other	1 (1.0)	0 (0.0)	16 (7.1)	19 (8.3)	0 (0.0)	3 (5.4)
Mean (SD) weight, kg[a]	65.6 (16.4)	69.4 (17.0)	79.0 (15.5)	79.1 (16.7)	77.2 (28.6)	66.2 (17.9)
Mean (SD) BMI, kg/m^2[b]	23.9 (4.9)	24.7 (5.2)	28.0 (5.6)	28.1 (5.1)	26.0 (8.7)	23.6 (5.1)
Mean (SD) APACHE II score[c]	6.1 (4.3)	6.3 (4.2)	–	–	14.8 (4.0)	15.8 (4.7)
APACHE II score grouping[c]						
<10	89 (85.6)	102 (78.5)	–	–	1 (1.9)	0 (0.0)
10–19	12 (11.5)	25 (19.2)	–	–	45 (83.3)	42 (72.4)
20–30	2 (1.9)	1 (0.8)	–	–	8 (14.8)	16 (27.6)
Missing/not done	1 (1.0)	2 (1.5)	231 (100)	232 (100)	0 (0.0)	0 (0.0)
CrCL (mL/min), *n* (%)						
≤30	0 (0.0)	2 (1.5)	6 (2.6)	6 (2.6)	1 (1.9)	0 (0.0)
31–50	9 (8.7)	11 (8.5)	32 (13.9)	30 (12.9)	5 (9.3)	2 (3.4)
51–80	28 (26.9)	29 (22.3)	91 (39.4)	85 (36.6)	17 (31.5)	24 (41.4)
≥81	66 (63.5)	88 (67.7)	102 (44.2)	111 (47.8)	31 (57.4)	31 (53.4)

Missing/not done	1 (1.0)	0 (0.0)	0 (0.0)	0 (0.0)	0 (0.0)	1 (1.7)
Primary diagnosis, *n* (%)						
VAP	0 (0.0)	0 (0.0)	0 (0.0)	0 (0.0)	29 (53.7)	28 (48.3)
NP (not associated with ventilator)	0 (0.0)	0 (0.0)	0 (0.0)	0 (0.0)	25 (46.3)	30 (51.7)
Acute pyelonephritis	0 (0.0)	0 (0.0)	101 (43.7)	116 (50.0)	0 (0.0)	0 (0.0)
cUTI without acute pyelonephritis	0 (0.0)	0 (0.0)	130 (56.3)	116 (50.0)	0 (0.0)	0 (0.0)
Traumatic perforation	3 (2.9)	1 (0.8)	0 (0.0)	0 (0.0)	0 (0.0)	0 (0.0)
Diverticular disease	5 (4.8)	7 (5.4)	0 (0.0)	0 (0.0)	0 (0.0)	0 (0.0)
Secondary peritonitis	13 (12.5)	10 (7.7)	0 (0.0)	0 (0.0)	0 (0.0)	0 (0.0)
Intra-abdominal abscess	12 (11.5)	15 (11.5)	0 (0.0)	0 (0.0)	0 (0.0)	0 (0.0)
Acute gastric and duodenal perforations	17 (16.3)	15 (11.5)	0 (0.0)	0 (0.0)	0 (0.0)	0 (0.0)
Cholecystitis	14 (13.5)	30 (23.1)	0 (0.0)	0 (0.0)	0 (0.0)	0 (0.0)
Appendiceal perforation or peri-appendiceal abscess	40 (38.5)	52 (40.0)	0 (0.0)	0 (0.0)	0 (0.0)	0 (0.0)
Infection type, *n* (%)						
Monomicrobial	60 (58.3)	86 (67.2)	219 (97.3)	222 (96.9)	28 (54.9)	31 (55.4)
Polymicrobial	43 (41.7)	42 (32.8)	6 (2.7)	7 (3.1)	23 (45.1)	25 (44.6)
2 pathogens	27 (26.2)	25 (19.5)	5 (2.2)	7 (3.1)	16 (31.4)	18 (32.1)
3 pathogens[d]	7 (6.8)	10 (7.8)	1 (0.4)	0 (0.0)	5 (9.8)	3 (5.4)
4 pathogens	4 (3.9)	6 (4.7)	0 (0.0)	0 (0.0)	2 (3.9)	4 (7.1)
≥5 pathogens	5 (4.9)	1 (0.8)	0 (0.0)	0 (0.0)	0 (0.0)	0 (0.0)

[a]Weight ‘Not done/missing’ for 1 patient with NP, including VAP (ceftazidime/avibactam, *n*=1).

[b]BMI ‘Not done/missing’ for 3 patients with cUTI (ceftazidime/avibactam, *n*=2; comparator, *n*=1) and 1 patient with NP, including VAP (ceftazidime/avibactam, *n*=1).

[c]APACHE II score calculated programmatically using data obtained at the site and reported in the electronic case report form for patients with cIAI and NP/VAP only. Data not collected for patients with cUTI. APACHE II score ‘Not done/missing’ for 3 patients with cIAI (ceftazidime/avibactam, *n*=1; comparator, *n*=2).

[d]A maximum of 2 uropathogens was allowed for study inclusion in the cUTI studies; however 1 patient randomized to the ceftazidime/avibactam group in REPRISE presented with 3 pathogens at baseline (*Proteus mirabilis* in the urine culture and 2 anaerobes in the blood culture).

cIAI, complicated intra-abdominal infection; CrCL, creatinine clearance; cUTI, complicated urinary tract infection; mMITT, microbiological modified intent-to-treat; NP, nosocomial pneumonia; SD, standard deviation; VAP, ventilator-associated pneumonia.

Table S3. Gram-negative pathogens identified in patients with β-lactamase-producing (non MBL) pathogens (mMITT population)

	cIAI		cUTI		NP, including VAP		All indications comb	
n (%)	Ceftazidime/avibactam + metronidazole (*n*=103)	Comparator (*n*=128)	Ceftazidime/avibactam (*n*=225)	Comparator (*n*=229)	Ceftazidime/avibactam (*n*=51)	Meropenem (*n*=56)	Ceftazidime/avibactam (*n*=379)	C
**Enterobacterales (all)**	**101 (98.1)**	**127 (99.2)**	**208 (92.4)**	**219 (95.6)**	**47 (92.2)**	**49 (87.5)**	**356 (93.9)**	
Citrobacter freundii complex	1 (1.0)	5 (3.9)	6 (2.7)	2 (0.9)	0 (0.0)	0 (0.0)	7 (1.8)	
Enterobacter aerogenes	0 (0.0)	1 (0.8)	1 (0.4)	0 (0.0)	4 (7.8)	2 (3.6)	5 (1.3)	
Enterobacter cloacae	7 (6.8)	8 (6.3)	13 (5.8)	13 (5.7)	10 (19.6)	7 (12.5)	30 (7.9)	
Escherichia coli	75 (72.8)	96 (75.0)	106 (47.1)	105 (45.9)	6 (11.8)	9 (16.1)	187 (49.3)	
Klebsiella oxytoca	3 (2.9)	1 (0.8)	1 (0.4)	2 (0.9)	0 (0.0)	0 (0.0)	4 (1.1)	
Klebsiella ozaenae	0 (0.0)	0 (0.0)	0 (0.0)	1 (0.4)	0 (0.0)	0 (0.0)	0 (0.0)	
Klebsiella pneumoniae	27 (26.2)	21 (16.4)	74 (32.9)	94 (41.0)	30 (58.8)	32 (57.1)	131 (34.6)	
Morganella morganii	1 (1.0)	1 (0.8)	1 (0.4)	0 (0.0)	0 (0.0)	1 (1.8)	2 (0.5)	
Proteus mirabilis	3 (2.9)	3 (2.3)	9 (4.0)	6 (2.6)	6 (11.8)	5 (8.9)	18 (4.7)	
Proteus vulgaris group	1 (1.0)	0 (0.0)	0 (0.0)	0 (0.0)	0 (0.0)	0 (0.0)	1 (0.3)	
Providencia rettgeri	0 (0.0)	0 (0.0)	1 (0.4)	1 (0.4)	0 (0.0)	0 (0.0)	1 (0.3)	
Raoultella terrigena	0 (0.0)	0 (0.0)	0 (0.0)	1 (0.4)	0 (0.0)	0 (0.0)	0 (0.0)	
Serratia marcescens	2 (1.9)	1 (0.8)	0 (0.0)	3 (1.3)	3 (5.9)	5 (8.9)	5 (1.3)	

**Other Gram-negative pathogens (all)**	**11 (10.7)**	**12 (9.4)**	**18 (8.0)**	**10 (4.4)**	**15 (29.4)**	**21 (37.5)**	**44 (11.6)**
Alcaligenes faecalis	1 (1.0)	2 (1.6)	0 (0.0)	0 (0.0)	0 (0.0)	0 (0.0)	1 (0.3)
Burkholderia cepacia complex	0 (0.0)	0 (0.0)	0 (0.0)	0 (0.0)	0 (0.0)	1 (1.8)	0 (0.0)
Comamonas testosteroni	1 (1.0)	0 (0.0)	0 (0.0)	0 (0.0)	0 (0.0)	0 (0.0)	1 (0.3)
Delftia acidovorans	1 (1.0)	0 (0.0)	0 (0.0)	0 (0.0)	0 (0.0)	0 (0.0)	1 (0.3)
Haemophilus influenzae	0 (0.0)	0 (0.0)	0 (0.0)	0 (0.0)	0 (0.0)	1 (1.8)	0 (0.0)
Haemophilus parainfluenzae	0 (0.0)	0 (0.0)	0 (0.0)	0 (0.0)	0 (0.0)	1 (1.8)	0 (0.0)
Pseudomonas aeruginosa	7 (6.8)	12 (9.4)	18 (8.0)	10 (4.4)	15 (29.4)	19 (33.9)	40 (10.6)
Pseudomonas otitidis	1 (1.0)	0 (0.0)	0 (0.0)	0 (0.0)	0 (0.0)	0 (0.0)	1 (0.3)

Patients could have ≥1 pathogen. Multiple isolates of the same species from the same patient are counted only once. Percentages are based on the total number of patients in the treatment group.

cIAI, complicated intra-abdominal infection; cUTI, complicated urinary tract infection; mMITT, microbiological modified intent-to-treat; NP, nosocomial pneumonia; TOC, test of cure; VAP, ventilator-associated pneumonia.

Table S4. β-lactamase status of baseline Gram-negative pathogens identified in patients with β-lactamase-producing (non-MBL) pathogens (pooled mMITT population)

	All patients		≥1 β-lactamase identified		No β-lactamase identified	
	Ceftazidime/avibactam (*n*=379)	Comparator (*n*=413)	Ceftazidime/avibactam (*n*=379)	Comparator (*n*=413)	Ceftazidime/avibactam (*n*=379)	Comparator (*n*=413)
**Enterobacterales (all)**	**356 (93.9)**	**395 (95.6)**	**354**	**393**	**25**	**25**
Citrobacter freundii complex	7 (1.8)	7 (1.7)	7	6	0	1
Enterobacter aerogenes	5 (1.3)	3 (0.7)	5	3	0	0
Enterobacter cloacae	30 (7.9)	28 (6.8)	27	27	3	1
Escherichia coli	187 (49.3)	210 (50.8)	182	199	5	11
Klebsiella oxytoca	4 (1.1)	3 (0.7)	1	3	3	0
Klebsiella ozaenae	0 (0.0)	1 (0.2)	0	0	0	1
Klebsiella pneumoniae	131 (34.6)	147 (35.6)	120	141	11	6
Morganella morganii	2 (0.5)	2 (0.5)	0	1	2	1
Proteus mirabilis	18 (4.7)	14 (3.4)	15	13	3	1
Proteus vulgaris group	1 (0.3)	0 (0.0)	0	0	1	0
Providencia rettgeri	1 (0.3)	1 (0.2)	1	1	0	0
Raoultella terrigena	0 (0.0)	1 (0.2)	0	0	0	1
Serratia marcescens	5 (1.3)	9 (2.2)	5	7	0	2
**Other Gram-negative pathogens (all)**	**44 (11.6)**	**43 (10.4)**	**26**	**27**	**18**	**18**
Alcaligenes faecalis	1 (0.3)	2 (0.5)	0	0	1	2
Burkholderia cepacia complex	0 (0.0)	1 (0.2)	0	0	0	1
Comamonas testosteroni	1 (0.3)	0 (0.0)	0	0	1	0

Delftia acidovorans	1 (0.3)	0 (0.0)	0	0	1	0
Haemophilus influenzae	0 (0.0)	1 (0.2)	0	0	0	1
Haemophilus parainfluenzae	0 (0.0)	1 (0.2)	0	0	0	1
Pseudomonas aeruginosa	40 (10.6)	41 (9.9)	26	27	14	14
Pseudomonas otitidis	1 (0.3)	0 (0.0)	0	0	1	0

Table S5. Number of β-lactamase enzymes and/or resistance mechanisms per isolate identified in β-lactamase-producing (non MBL) baseline Gram-negative isolates (pooled mMITT population)

	Number of β-lactamase enzymes	Ceftazidime/avibactam (*n*=379)	Comparator (*n*=413)
**Enterobacterales (all)**	1	16 (4.2)	12 (2.9)
	2	61 (16.1)	58 (14.0)
	≥3	281 (74.1)	326 (78.9)
Citrobacter freundii complex	1	1 (0.3)	2 (0.5)
	≥3	6 (1.6)	4 (1.0)
Enterobacter aerogenes	1	3 (0.8)	2 (0.5)
	2	1 (0.3)	0 (0.0)
	≥3	1 (0.3)	1 (0.2)
Enterobacter cloacae	1	8 (2.1)	5 (1.2)
	2	2 (0.5)	2 (0.5)
	≥3	17 (4.5)	20 (4.8)
Escherichia coli	1	2 (0.5)	2 (0.5)
	2	48 (12.7)	48 (11.6)
	≥3	132 (34.8)	149 (36.1)
Klebsiella oxytoca	≥3	1 (0.3)	3 (0.7)
Klebsiella pneumoniae	1	0 (0.0)	1 (0.2)
	2	5 (1.3)	2 (0.5)
	≥3	115 (30.3)	138 (33.4)
Morganella morganii	≥3	0 (0.0)	1 (0.2)
Proteus mirabilis	2	4 (1.1)	2 (0.5)
	≥3	11 (2.9)	11 (2.7)
Providencia rettgeri	2	0 (0.0)	1 (0.2)
	≥3	1 (0.3)	0 (0.0)
Serratia marcescens	1	2 (0.5)	0 (0.0)
	2	1 (0.3)	3 (0.7)
	≥3	2 (0.5)	4 (1.0)
**Other Gram-negative pathogens (all)**	1	11 (2.9)	10 (2.4)
	2	3 (0.8)	6 (1.5)
	≥3	12 (3.2)	11 (2.7)
Pseudomonas aeruginosa	1	11 (2.9)	10 (2.4)

	2	3 (0.8)	6 (1.5)
	≥3	12 (3.2)	11 (2.7)

Patients could have ≥1 pathogen. Multiple isolates of the same species from the same patient are counted only once. Percentages are based on the total number of patients in the treatment group.

mMITT, microbiological modified intent-to-treat.

Table S6. β-lactamase genes and β-lactam resistance mechanisms identified in β-lactamase-producing (non MBL) pathogens (pooled mMITT population)

Baseline pathogen	β-lactamase / β-lactam resistance mechanisms	Ceftazidime/avibactam (*n*=379)	Comparator (*n*=413)
Citrobacter freundii complex		7 (1.8)	6 (1.5)
	AmpC Overexpression	4 (1.1)	5 (1.2)
	Cmy-86-Like	1 (0.3)	0 (0.0)
	Cmy-Like	1 (0.3)	0 (0.0)
	Ctx-M-15-Like	4 (1.1)	3 (0.7)
	Ctx-M-3-Like	2 (0.5)	1 (0.2)
	Ctx-M-Like	6 (1.6)	3 (0.7)
	Dha-4	1 (0.3)	0 (0.0)
	Dha-Like	1 (0.3)	0 (0.0)
	Oxa-1/30	3 (0.8)	3 (0.7)
	Oxa-Like	3 (0.8)	3 (0.7)
	Tem-1	6 (1.6)	4 (1.0)
	Tem-Like	6 (1.6)	4 (1.0)
Enterobacter aerogenes		5 (1.3)	3 (0.7)
	AmpC Overexpression	3 (0.8)	2 (0.5)
	Ctx-M-14	1 (0.3)	0 (0.0)
	Ctx-M-15-Like	1 (0.3)	0 (0.0)
	Ctx-M-3	1 (0.3)	0 (0.0)
	Ctx-M-3-Like	0 (0.0)	1 (0.2)
	Ctx-M-Like	2 (0.5)	1 (0.2)
	Tem-1	1 (0.3)	1 (0.2)
	Tem-Like	1 (0.3)	1 (0.2)
Enterobacter cloacae		27 (7.1)	27 (6.5)
	Act-Like	1 (0.3)	0 (0.0)
	AmpC Overexpression	19 (5.0)	18 (4.4)
	Ctx-M-15-Like	10 (2.6)	15 (3.6)
	Ctx-M-3-Like	4 (1.1)	4 (1.0)
	Ctx-M-Like	14 (3.7)	17 (4.1)
	Dha-1	1 (0.3)	0 (0.0)
	Dha-Like	1 (0.3)	1 (0.2)

	Mir-Like	1 (0.3)	0 (0.0)
	Oxa-1/30	11 (2.9)	14 (3.4)
	Oxa-Like	11 (2.9)	15 (3.6)
	Shv-12	5 (1.3)	3 (0.7)
	Shv-Like	5 (1.3)	3 (0.7)
	Tem-1	14 (3.7)	16 (3.9)
	Tem-Like	15 (4.0)	16 (3.9)
Escherichia coli		182 (48.0)	199 (48.2)
	Act-24	1 (0.3)	0 (0.0)
	Act-Like	1 (0.3)	0 (0.0)
	AmpC Overexpression	1 (0.3)	2 (0.5)
	Cmy-2	4 (1.1)	5 (1.2)
	Cmy-2-Like	5 (1.3)	8 (1.9)
	Cmy-4	1 (0.3)	0 (0.0)
	Cmy-42	5 (1.3)	3 (0.7)
	Cmy-42-Like	2 (0.5)	0 (0.0)
	Cmy-59	1 (0.3)	0 (0.0)
	Cmy-Like	17 (4.5)	16 (3.9)
	Ctx-M-1	1 (0.3)	1 (0.2)
	Ctx-M-1-Like	5 (1.3)	2 (0.5)
	Ctx-M-138	1 (0.3)	0 (0.0)
	Ctx-M-14	18 (4.7)	27 (6.5)
	Ctx-M-142	0 (0.0)	1 (0.2)
	Ctx-M-15	3 (0.8)	6 (1.5)
	Ctx-M-15-Like	111 (29.3)	130 (31.5)
	Ctx-M-2	0 (0.0)	2 (0.5)
	Ctx-M-24	0 (0.0)	2 (0.5)
	Ctx-M-27	22 (5.8)	13 (3.1)
	Ctx-M-3	1 (0.3)	1 (0.2)
	Ctx-M-3-Like	3 (0.8)	7 (1.7)
	Ctx-M-32	0 (0.0)	1 (0.2)
	Ctx-M-55/57	5 (1.3)	8 (1.9)
	Ctx-M-55/79	2 (0.5)	6 (1.5)
	Ctx-M-65	1 (0.3)	0 (0.0)
	Ctx-M-9	0 (0.0)	1 (0.2)
	Ctx-M-Like	163 (43.0)	184 (44.6)
	Dha-1	3 (0.8)	2 (0.5)
	Dha-Like	3 (0.8)	2 (0.5)
	Lap-2	0 (0.0)	1 (0.2)

	Oxa-1	2 (0.5)	3 (0.7)
	Oxa-1/30	67 (17.7)	83 (20.1)
	Oxa-1/30-Like	1 (0.3)	0 (0.0)
	Oxa-2	1 (0.3)	1 (0.2)
	Oxa-Like	71 (18.7)	88 (21.3)
	Shv-12	2 (0.5)	1 (0.2)
	Shv-2	0 (0.0)	1 (0.2)
	Shv-Like	2 (0.5)	2 (0.5)
	Tem-1	80 (21.1)	82 (19.9)
	Tem-1-Like	2 (0.5)	1 (0.2)
	Tem-135	0 (0.0)	1 (0.2)
	Tem-176	1 (0.3)	0 (0.0)
	Tem-33-Like	0 (0.0)	1 (0.2)
	Tem-40	0 (0.0)	2 (0.5)
	Tem-Like	83 (21.9)	88 (21.3)
Klebsiella oxytoca		1 (0.3)	3 (0.7)
	Ctx-M-14	0 (0.0)	1 (0.2)
	Ctx-M-15-Like	0 (0.0)	2 (0.5)
	Ctx-M-Like	0 (0.0)	3 (0.7)
	Lap-2	0 (0.0)	1 (0.2)
	Oxa-1/30	0 (0.0)	1 (0.2)
	Oxa-48	1 (0.3)	0 (0.0)
	Oxa-48-Like	1 (0.3)	0 (0.0)
	Oxa-9-Like	0 (0.0)	1 (0.2)
	Oxa-Like	0 (0.0)	2 (0.5)
	Shv-11-Like	0 (0.0)	1 (0.2)
	Shv-Like	0 (0.0)	1 (0.2)
	Tem-1	1 (0.3)	2 (0.5)
	Tem-15	0 (0.0)	1 (0.2)
	Tem-Like	1 (0.3)	3 (0.7)
Klebsiella pneumoniae		120 (31.7)	141 (34.1)
	Cmy-4	0 (0.0)	2 (0.5)
	Cmy-Like	0 (0.0)	2 (0.5)
	Ctx-M-14	2 (0.5)	4 (1.0)
	Ctx-M-15	0 (0.0)	1 (0.2)
	Ctx-M-15-Like	95 (25.1)	109 (26.4)
	Ctx-M-2	1 (0.3)	0 (0.0)
	Ctx-M-27	2 (0.5)	0 (0.0)
	Ctx-M-3	2 (0.5)	4 (1.0)

Ctx-M-3-Like	5 (1.3)	9 (2.2)
Ctx-M-55/57	0 (0.0)	1 (0.2)
Ctx-M-65	1 (0.3)	3 (0.7)
Ctx-M-9	3 (0.8)	0 (0.0)
Ctx-M-Like	109 (28.8)	124 (30.0)
Dha-1	5 (1.3)	5 (1.2)
Dha-Like	5 (1.3)	5 (1.2)
Kpc-2	2 (0.5)	4 (1.0)
Kpc-3	2 (0.5)	2 (0.5)
Kpc-Like	4 (1.1)	6 (1.5)
Lap-2	1 (0.3)	1 (0.2)
Okp-A	1 (0.3)	0 (0.0)
Oxa-1	0 (0.0)	2 (0.5)
Oxa-1/30	83 (21.9)	82 (19.9)
Oxa-1/30-Like	1 (0.3)	2 (0.5)
Oxa-10	2 (0.5)	2 (0.5)
Oxa-2	1 (0.3)	2 (0.5)
Oxa-48	4 (1.1)	2 (0.5)
Oxa-48-Like	4 (1.1)	2 (0.5)
Oxa-9-Like	0 (0.0)	5 (1.2)
Oxa-Like	88 (23.2)	93 (22.5)
Shv-1	40 (10.6)	56 (13.6)
Shv-1-Like	13 (3.4)	12 (2.9)
Shv-107	1 (0.3)	3 (0.7)
Shv-11	43 (11.3)	46 (11.1)
Shv-11-Like	8 (2.1)	13 (3.1)
Shv-110	1 (0.3)	0 (0.0)
Shv-115	1 (0.3)	0 (0.0)
Shv-12	2 (0.5)	5 (1.2)
Shv-18	1 (0.3)	2 (0.5)
Shv-2	0 (0.0)	1 (0.2)
Shv-2/-106	0 (0.0)	1 (0.2)
Shv-26	0 (0.0)	1 (0.2)
Shv-27	0 (0.0)	1 (0.2)
Shv-28	0 (0.0)	1 (0.2)
Shv-31	1 (0.3)	0 (0.0)
Shv-33	2 (0.5)	2 (0.5)
Shv-38	2 (0.5)	1 (0.2)
Shv-5	2 (0.5)	1 (0.2)

	Shv-5/-55/-131	0 (0.0)	1 (0.2)
	Shv-71	1 (0.3)	1 (0.2)
	Shv-75	1 (0.3)	0 (0.0)
	Shv-76	1 (0.3)	1 (0.2)
	Shv-Like	118 (31.1)	140 (33.9)
	Tem-1	81 (21.4)	86 (20.8)
	Tem-1-Like	11 (2.9)	15 (3.6)
	Tem-209	1 (0.3)	0 (0.0)
	Tem-79	0 (0.0)	1 (0.2)
	Tem-Like	93 (24.5)	102 (24.7)
Morganella morganii		0 (0.0)	1 (0.2)
	Ctx-M-14	0 (0.0)	1 (0.2)
	Ctx-M-Like	0 (0.0)	1 (0.2)
	Tem-1	0 (0.0)	1 (0.2)
	Tem-Like	0 (0.0)	1 (0.2)
Proteus mirabilis		15 (4.0)	13 (3.1)
	Acc-4	3 (0.8)	3 (0.7)
	Acc-Like	3 (0.8)	3 (0.7)
	Cmy-12	0 (0.0)	1 (0.2)
	Cmy-15	1 (0.3)	0 (0.0)
	Cmy-16	3 (0.8)	2 (0.5)
	Cmy-16-Like	1 (0.3)	1 (0.2)
	Cmy-2	3 (0.8)	0 (0.0)
	Cmy-2-Like	1 (0.3)	0 (0.0)
	Cmy-22	1 (0.3)	0 (0.0)
	Cmy-Like	8 (2.1)	4 (1.0)
	Ctx-M-116/-136	1 (0.3)	0 (0.0)
	Ctx-M-14	2 (0.5)	1 (0.2)
	Ctx-M-15-Like	0 (0.0)	1 (0.2)
	Ctx-M-3-Like	0 (0.0)	1 (0.2)
	Ctx-M-44	0 (0.0)	1 (0.2)
	Ctx-M-55/79	1 (0.3)	0 (0.0)
	Ctx-M-Like	4 (1.1)	4 (1.0)
	Dha-1	0 (0.0)	1 (0.2)
	Dha-Like	0 (0.0)	1 (0.2)
	Oxa-1/30	0 (0.0)	2 (0.5)
	Oxa-2	1 (0.3)	1 (0.2)
	Oxa-9-Like	0 (0.0)	1 (0.2)
	Oxa-Like	1 (0.3)	4 (1.0)

	Shv-12	0 (0.0)	1 (0.2)
	Shv-5/-55/-131	0 (0.0)	1 (0.2)
	Shv-Like	0 (0.0)	2 (0.5)
	Tem-1	10 (2.6)	11 (2.7)
	Tem-1-Like	0 (0.0)	1 (0.2)
	Tem-2	1 (0.3)	2 (0.5)
	Tem-Like	11 (2.9)	13 (3.1)
	Veb-6	1 (0.3)	0 (0.0)
Providencia rettgeri		1 (0.3)	1 (0.2)
	Per-1	1 (0.3)	0 (0.0)
	Tem-1	1 (0.3)	1 (0.2)
	Tem-Like	1 (0.3)	1 (0.2)
Serratia marcescens		5 (1.3)	7 (1.7)
	AmpC Overexpression	2 (0.5)	0 (0.0)
	Cmy-4-Like	0 (0.0)	1 (0.2)
	Cmy-Like	0 (0.0)	1 (0.2)
	Ctx-M-14	1 (0.3)	3 (0.7)
	Ctx-M-15-Like	1 (0.3)	2 (0.5)
	Ctx-M-3	0 (0.0)	1 (0.2)
	Ctx-M-3-Like	1 (0.3)	2 (0.5)
	Ctx-M-Like	3 (0.8)	7 (1.7)
	Oxa-1/30	1 (0.3)	2 (0.5)
	Oxa-Like	1 (0.3)	2 (0.5)
	Tem-1	2 (0.5)	4 (1.0)
	Tem-Like	2 (0.5)	4 (1.0)
Pseudomonas aeruginosa		26 (6.9)	27 (6.5)
	AmpC Overexpression	17 (4.5)	17 (4.1)
	Ctx-M-15-Like	0 (0.0)	1 (0.2)
	Ctx-M-3	0 (0.0)	1 (0.2)
	Ctx-M-Like	0 (0.0)	1 (0.2)
	Mexab-Oprm_ overexpression_ interp	0 (0.0)	1 (0.2)
	Mexcd-Oprj_ overexpression_ interp	0 (0.0)	2 (0.5)
	Mexxy-Oprm_ overexpression_ interp	1 (0.3)	1 (0.2)
	Oprd_loss	1 (0.3)	3 (0.7)
	Oxa-10	5 (1.3)	1 (0.2)
	Oxa-17	1 (0.3)	0 (0.0)
	Oxa-2	5 (1.3)	8 (1.9)
	Oxa-74	3 (0.8)	0 (0.0)
	Oxa-Like	12 (3.2)	9 (2.2)

Per-1	2 (0.5)	6 (1.5)
Per-1-Like	1 (0.3)	0 (0.0)
Per-Like	3 (0.8)	6 (1.5)
Pse-Like	2 (0.5)	0 (0.0)
Tem-1	1 (0.3)	2 (0.5)
Tem-Like	1 (0.3)	2 (0.5)
Veb-1-Like	1 (0.3)	0 (0.0)
Veb-9	3 (0.8)	1 (0.2)
Veb-Like	4 (1.1)	1 (0.2)

Patients could have ≥1 pathogen. Multiple isolates of the same species from the same patient are counted only once. Percentages are based on the total number of patients in the treatment group.

mMITT, microbiological modified intent-to-treat.

Table S7. Ceftazidime/avibactam minimum inhibitory concentrations for Enterobacterales and *P. aeruginosa* isolates from patients with β-lactamase-producing (non-MBL) Gram-negative pathogens identified at baseline (pooled mMITT population)[a,b]

Baseline pathogen	Treatment group	Number of isolates tested	MIC data, mg/L			Susceptible %[c]
			Range	MIC_{50}	MIC_{90}	
All Enterobacterales	Ceftazidime/avibactam	390	≤0.008 – 8	0.25	1	100
	Comparator	421	≤0.008 –32	0.25	1	99.8
	Total	811	≤0.008 –32	0.25	1	99.9
Enterobacter cloacae	Ceftazidime/avibactam	30	0.12 – 4	0.5	2	100
	Comparator	28	0.12 – 4	0.5	2	100
	Total	58	0.12 – 4	0.5	2	100
Escherichia coli	Ceftazidime/avibactam	187	≤0.008 – 8	0.12	0.5	100
	Comparator	206	≤0.008 – 8	0.12	0.5	100
	Total	393	≤0.008 – 8	0.12	0.5	100
Klebsiella pneumoniae	Ceftazidime/avibactam	131	0.03 – 4	0.5	1	100
	Comparator	147	0.03 – 4	0.5	1	100
	Total	278	0.03 – 4	0.5	1	100
Pseudomonas aeruginosa	Ceftazidime/avibactam	40	1 – >32	2	>32	72.5
	Comparator	41	1 – >32	4	32	82.5
	Total	81	1 – >32	4	32	76.5

Patients could have ≥1 pathogen. Multiple isolates of the same species from the same patient are counted only once.

[a]For pathogens with n≥10 isolates tested in either treatment group.

[b]Includes all pathogens isolated from patients with β-lactamase isolates (including non-β-lactamase producing pathogens).

[c]Based on EUCAST and CLSI MIC susceptible breakpoints i.e. MIC ≤8 mg/L.

mMITT, microbiological modified intent-to-treat.

Table S8. Clinical cure rates at TOC by baseline pathogen in patients with β-lactamase-producing (non-MBL) Gram-negative pathogens identified at baseline (pooled mMITT population)

	Ceftazidime/avibactam (*n*=379)		Comparator (*n*=413)	
	**n/N (%)**	**95% CI[a]**	**n/N (%)**	**95% CI[a]**
**Enterobacterales (all)**	**315/356 (88.5)**	**84.6, 91.1**	**347/295 (87.8)**	**84.8, 91.0**
Citrobacter freundii complex	6/7 (85.7)	49.9, 98.4	6/7 (85.7)	49.9, 98.4
Enterobacter aerogenes	4/5 (80.0)	37.1, 97.7	3/3 (100)	46.4,100.0
Enterobacter cloacae	26/30 (86.7)	71.3, 95.3	20/28 (71.4)	53.2, 85.5
Escherichia coli	163/187 (87.2)	81.8, 91.4	183/210 (87.1)	82.1, 91.2
Klebsiella oxytoca	4/4 (100)	55.5,100	3/3 (100)	46.4, 100
Klebsiella ozaenae	0/0	–	1/1 (100)	14.7, 100
Klebsiella pneumoniae	118/131 (90.1)	84.1, 94.3	130/147 (88.4)	82.5, 92.8
Morganella morganii	2/2(100)	33.3, 100	1/2 (50.0)	6.1, 93.9
Proteus mirabilis	16/18 (88.9)	68.9, 97.6	13/14 (92.9)	71.2, 99.2
Proteus vulgaris group	1/1 (100)	14.7, 100	0/0	–
Providencia rettgeri	1/1 (100)	14.7, 100	1/1 (100)	14.7, 100
Raoultella terrigena	0/0	–	1/1 (100)	14.7, 100
Serratia marcescens	4/5 (80.0)	37.1, 97.7	8/9 (88.9)	58.6, 98.8
**Other Gram-negative pathogens (all)**	**9/12 (75.0)**	**47.1, 92.4**	**13/15 (86.7)**	**63.7, 97.1**
Alcaligenes faecalis	1/1 (100)	14.7, 100	2/2 (100)	33.3, 100
Burkholderia cepacia complex	0/0	–	0/1 (0.0)	0.0, 85.3
Comamonas testosteroni	1/1 (100)	14.7, 100	0/0	–
Delftia acidovorans	1/1 (100)	14.7, 100	0/0	–
Haemophilus influenzae	0/0	–	0/1 (0.0)	0.0, 85.3
Haemophilus parainfluenzae	0/0	–	1/1 (100)	14.7, 100
Pseudomonas aeruginosa	30/40 (75.0)	60.2, 86.4	36/41 (87.8)	75.3, 95.2
Pseudomonas otitidis	1/1 (100)	14.7, 100	0/0	–

Patients could have ≥1 pathogen. Multiple isolates of the same species from the same patient are counted only once.

[a]Calculated using the Jeffreys method.

CI, confidence interval; mMITT, microbiological modified intent-to-treat; N, number of patients in the treatment arm with a pathogen of the specified type; n, number of patients with clinical cure; TOC, test of cure.

Table S9. Favourable microbiological response rates at TOC by baseline pathogen in patients with β-lactamase-producing (non MBL) Gram-negative pathogens identified at baseline (mMITT population)

	**cIAI**		**cUTI/pyelonephritis**		**NP, including VAP**		**All indications combined**	
	**Ceftazidime/avibactam + metronidazole (*n*=103)**	**Comparator (*n*=128)**	**Ceftazidime/avibactam (*n*=225)**	**Comparator (*n*=229)**	**Ceftazidime/avibactam (*n*=51)**	**Meropenem (*n*=56)**	**Ceftazidime/avibactam (*n*=379)**	**Comparator (*n*=413)**
**Enterobacterales**								
Citrobacter freundii complex	0/1 (0.0)	4/5 (80.0)	5/6 (83.3)	0/2 (0.0)	0/0	0/0	5/7 (71.4)	4/7 (57.1)
Enterobacter aerogenes	0/0	1/1 (100)	1/1 (100)	0/0	3/4 (75.0)	2/2 (100)	4/5 (80.0)	3/3 (100)
Enterobacter cloacae	6/7 (85.7)	6/8 (75.0)	8/13 (61.5)	10/13 (76.9)	8/10 (80.0)	5/7 (71.4)	22/30 (73.3)	21/28 (75.0)
Escherichia coli	62/75 (82.7)	83/96 (86.5)	83/106 (78.3)	64/103 (62.1)	5/6 (83.3)	7/9 (77.8)	150/187 (80.2)	154/208 (74.0)
Klebsiella oxytoca	3/3 (100)	1/1 (100)	0/1	1/2 (50.0)	0/0	0/0	3/4 (75.0)	2/3 (66.7)
Klebsiella ozaenae	0/0	0/0	0/0	1/1 (100)	0/0	0/0	0/0	1/1 (100)
Klebsiella pneumoniae	23/27 (85.2)	16/21 (76.2)	60/74 (81.1)	60/94 (63.8)	21/30 (70.0)	21/32 (65.6)	104/131 (79.4)	97/147 (66.0)
Morganella morganii	1/1 (100.0)	1/1 (100.0)	1/1 (100)	0/0	0/0	0/1	2/2 (100.0)	1/2 (50.0)
Proteus mirabilis	3/3 (100.0)	3/3 (100.0)	6/9 (66.7)	4/6 (66.7)	5/6 (83.3)	3/5 (60.0)	14/18 (77.8)	10/14 (71.4)
Proteus vulgaris group	1/1 (100)	0/0	0/0	0/0	0/0	0/0	1/1 (100)	0/0
Providencia rettgeri	0/0	0/0	0/1 (0.0)	0/1 (0.0)	0/0	0/0	0/1 (0.0)	0/1 (0.0)
Raoultella terrigena	0/0	0/0	0/0	1/1 (100)	0/0	0/0	0/0	1/1 (100)
Serratia marcescens	2/2 (100)	1/1 (00)	0/0	2/3 (66.7)	2/3 (66.7)	3/5 (60.0)	4/5 (80.0)	6/9 (66.7)
**Other Gram-negative pathogens**								
Alcaligenes faecalis	1/1 (100)	2/2 (100)	0/0	0/0	0/0	0/0	1/1 (100)	2/2 (100)
Burkholderia cepacia complex	0/0	0/0	0/0	0/0	0/0	0/1 (0.0)	0/0	0/1 (0.0)
Comamonas testosteroni	1/1 (100)	0/0	0/0	0/0	0/0	0/0	1/1 (100)	0/0
Delftia acidovorans	1/1 (100)	0/0	0/0	0/0	0/0	0/0	1/1 (100)	0/0
Haemophilus influenzae	0/0	0/0	0/0	0/0	0/0	0/1 (0.0)	0/0	0/1 (0.0)

Haemophilus parainfluenzae	0/0	0/0	0/0	0/0	0/0	1/1 (100)	0/0	1/1 (100)
Pseudomonas aeruginosa	3/7 (42.9)	12/12 (100.0)	13/18 (72.2)	7/10 (70.0)	5/15 (33.3)	6/19 (31.6)	21/40 (52.5)	25/41 (61.0)
Pseudomonas otitidis	1/1 (100)	0/0	0/0	0/0	0/0	0/0	1/1 (100)	0/0

Patients could have ≥1 pathogen. Multiple isolates of the same species from the same patient are counted only once.

cIAI, complicated intra-abdominal infection; cUTI, complicated urinary tract infection; mMITT, microbiological modified intent-to-treat; NP, nosocomial pneumonia; TOC, test of cure; VAP, ventilator-associated pneumonia.

Figure S1. Clinical cure rates at TOC in patients with β-lactamase producing (non-MBL) Gram-negative pathogens identified at baseline (pooled mMITT population)[a,b]

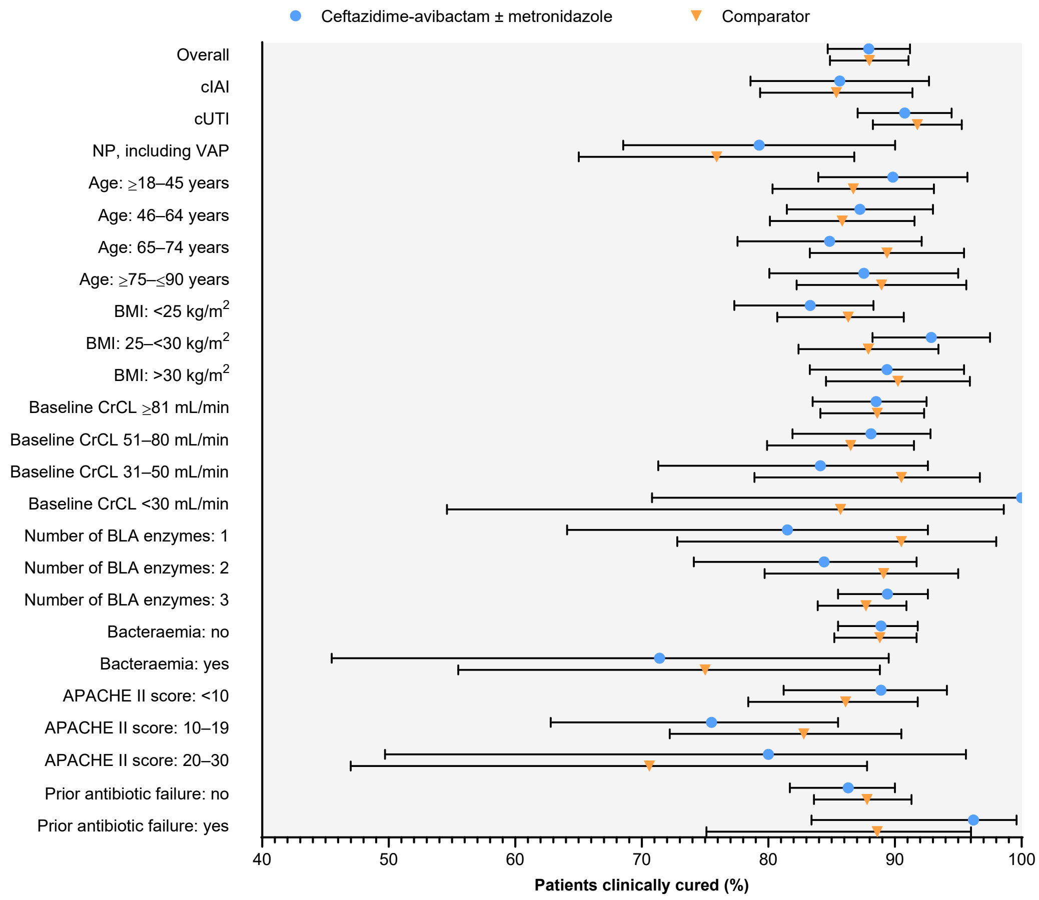

[a]BMI subgroups exclude two patients with cUTI with status ‘Not done/missing’ (ceftazidime/avibactam, *n*=1; comparator, *n*=1). CrCL subgroups exclude two patients with status ‘Not done/missing (ceftazidime/avibactam, *n*=1, cIAI group; comparator, *n*=1, NP including VAP group). APACHE II score subgroups exclude all patients with cUTI (ceftazidime/avibactam, *n*=225; comparator, *n*=229), and three cIAI patients with status ‘Not done/missing (ceftazidime/avibactam, *n*=1; comparator, *n*=2). Prior antibiotic subgroups exclude 185 patients with cUTI with status ‘Not done/missing’ (ceftazidime/avibactam, *n*=89; comparator, *n*=96) and five patients with NP, including VAP with status ‘Other’ (ceftazidime/avibactam, *n*=2; comparator, *n*=3).

[b]CIs calculated using the Jeffreys method.

BMI, body mass index; CI, confidence interval; cIAI, complicated intra-abdominal infection; CrCL, creatinine clearance; cUTI, complicated urinary tract infection; mMITT, microbiological modified intent-to-treat; NP, nosocomial pneumonia; TOC, test of cure; VAP, ventilator-associated pneumonia.

References

1. CLSI. Performance Standards for Antimicrobial Susceptibility Testing—Twenty-second Edition: M100. 2012.

2. Mazuski JE, Gasink LB, Armstrong J *et al.* Efficacy and safety of ceftazidime-avibactam plus metronidazole versus meropenem in the treatment of complicated intra-abdominal infection: results from a randomized, controlled, double-blind, phase 3 program. *Clin Infect Dis* 2016; **62**: 1380-9.

3. Qin X, Tran BG, Kim MJ *et al.* A randomised, double-blind, phase 3 study comparing the efficacy and safety of ceftazidime/avibactam plus metronidazole versus meropenem for complicated intra-abdominal infections in hospitalised adults in Asia. *Int J Antimicrob Agents* 2017; **49**: 579-88.

4. Carmeli Y, Armstrong J, Laud PJ *et al.* Ceftazidime-avibactam or best available therapy in patients with ceftazidime-resistant Enterobacteriaceae and *Pseudomonas aeruginosa* complicated urinary tract infections or complicated intra-abdominal infections (REPRISE): a randomised, pathogen-directed, phase 3 study. *Lancet Infect Dis* 2016; **16**: 661-73.

5. Wagenlehner FM, Sobel JD, Newell P *et al.* Ceftazidime-avibactam versus doripenem for the treatment of complicated urinary tract infections, including acute pyelonephritis: RECAPTURE, a phase 3 randomized trial program. *Clin Infect Dis* 2016; **63**: 754-62.

6. Torres A, Zhong N, Pachl J *et al.* Ceftazidime-avibactam versus meropenem in nosocomial pneumonia, including ventilator-associated pneumonia (REPROVE): a randomised, double-blind, phase 3 non-inferiority trial. *Lancet Infect Dis* 2018; **18**: 285-95.
